# HIV Care Through Their Eyes: Navigating HIV Services and ART Adherence Among Adolescents and Their Caregivers in Lusaka, Zambia

**DOI:** 10.1155/arat/4933427

**Published:** 2026-04-28

**Authors:** Kaala Moomba, Talitha Crowley, Brian Van Wyk

**Affiliations:** ^1^ Faculty of Community and Health Sciences, School of Public Health, University of the Western Cape, Cape Town, South Africa, uwc.ac.za; ^2^ Faculty of Community and Health Sciences, School of Nursing, University of the Western Cape, Cape Town, South Africa, uwc.ac.za

## Abstract

**Introduction:**

Adolescents living with HIV (ALHIV) face unique psychosocial and structural challenges affecting ART adherence and engagement in care. Despite Zambia’s expansion of adolescent‐friendly HIV services, adolescents continue to experience poorer adherence and treatment outcomes than adults, including lower viral suppression and retention in care and limited understanding remains around the experiences of ALHIV and their caregivers. This study explored multilevel factors influencing ART adherence in Lusaka district from the perspectives of ALHIV and their caregivers.

**Methods:**

We conducted an exploratory qualitative study on psychosocial, behavioral, and structural factors influencing ART adherence and experiences of HIV services among ALHIV in Lusaka, Zambia. Between February and July 2025, 35 in‐depth interviews (20 ALHIV who were already aware of their HIV status and engaged in long‐term care and 15 caregivers) were conducted across six health facilities (four PHC and two first‐level facilities). Interviews were held in English or Nyanja, audio‐recorded, transcribed, and analyzed using ATLAS.ti v24. A hybrid inductive‐deductive approach, informed by the socioecological model, guided coding. Themes were organized in an analytical matrix showing multilevel influences on adherence, including emotional burden, stigma, support systems, and structural barriers. Trustworthiness was ensured through triangulation, peer debriefing, and iterative codebook development.

**Results:**

The themes identified spanned intrapersonal (individual), interpersonal, organizational/institutional, community, and structural/policy‐level factors influencing ART adherence, alongside participant‐generated cross‐cutting recommendations to strengthen adolescent HIV service delivery. Many adolescents struggled with stigma, emotional distress, and challenges of consistent medication adherence, while others adapted through acceptance, spirituality, and counseling. Supportive relationships with family, peers, and healthcare workers fostered adherence, but stigma, school challenges, inconsistent caregiving, and limited privacy remained significant barriers. Socioeconomic issues, such as transport costs, hindered treatment continuity. Helpful strategies included routine‐building, reminders, and increased health literacy. Participants recommended improving adolescent‐friendly services by integrating mental health support, strengthening provider communication, expanding peer networks, and reactivating support groups to better support ART adherence among ALHIV.

**Conclusion:**

Addressing psychological, social, service delivery, and structural barriers such as coordination between schools and the health system is vital to improving ART adherence among ALHIV in Zambia. A multilevel approach is needed to support sustainable treatment outcomes.

## 1. Introduction

Adolescents living with HIV (ALHIV) represent a uniquely vulnerable population in the global HIV epidemic [1]. In 2024, approximately 370,000 young people aged 15 to 24 years were newly infected with HIV, including 145,000 adolescents between the ages of 15 and 19 years [2]. Individuals aged 15–24 years now account for one‐third of all new infections globally [3], and of the estimated 1.57 million adolescents aged 10 to 19 years living with HIV worldwide, only 64% (1.1 million) were receiving life‐saving antiretroviral therapy (ART) [4]. Sub‐Saharan Africa bears the greatest burden, with 78% of children and 83% of ALHIV located in the region [5]. In Zambia alone, an estimated 56,000 adolescents aged 10–19 years are living with HIV, with approximately 89% of them on ART, yet many still face gaps in testing and care engagement compared with adults [6]. Moreover, national impact assessments indicate that viral load suppression among HIV‐positive individuals aged 15–24 years is markedly lower (about 70.9%) than in older age groups (35–44 years; 88%), highlighting poorer treatment outcomes in this adolescent age band [7] While significant advances in access to ART have transformed HIV from a fatal illness to a manageable chronic condition, adolescents continue to experience disproportionately poor treatment outcomes compared to other age groups [8]. Despite efforts to scale up youth‐friendly health services, ALHIV remain at high risk of treatment interruption, poor adherence, virologic failure, and loss to follow‐up [9].

Adolescence is a complex developmental stage marked by physiological, psychological, and social transitions that can pose significant challenges to consistent engagement with long‐term chronic care [10]. For ALHIV, these challenges are often magnified by stigma, lack of autonomy, poor mental health, limited disclosure of HIV status, and inadequate support systems [11]. Stigma, both internalized and enacted, has been repeatedly cited as a key barrier to ART adherence among adolescents and often results in missed appointments or failure to collect medication [12, 13]. Moreover, fear of disclosure and peer rejection may lead adolescents to hide their status, avoid taking medication in public, or disengage from care altogether [14]. Adolescent adherence to ART is influenced by the complex interplay of psychosocial, behavioral, and socioeconomic factors, the nature of the treatment regimen, and the accessibility of healthcare services [15, 16]. Even after initiating ART, many adolescents remain unaware of their HIV serostatus or the underlying reasons for daily medication use, creating knowledge gaps that pose significant barriers to sustained adherence [16, 17].

Caregivers—defined broadly to include biological parents, extended family members, foster parents, and other adult guardians—play a pivotal role in the care continuum for ALHIV [18, 19]. Their involvement is particularly crucial in promoting adherence, facilitating clinic visits, providing psychosocial support and navigating disclosure processes [20]. Positive caregiver involvement has been shown to improve ART adherence and mental health outcomes, while caregiver stress, lack of knowledge, or overprotectiveness can hinder an adolescent’s transition toward self‐management [21]. Furthermore, the relationship between caregivers and health providers can influence adolescents’ experiences of healthcare and trust in the system [22].

However, caregivers also face numerous challenges. Many are themselves affected by poverty, HIV‐related stigma, and limited education about ART—all of which may constrain their ability to support adolescents effectively [23]. In cases where adolescents are orphaned or living with nonbiological caregivers, emotional attachment and continuity of care may be weakened, which further complicates adherence and retention in ART [24].

According to the most recent Zambia Demographic and Health Survey (ZDHS) conducted in 2018, the HIV prevalence among adolescents aged 15–19 years was reported at 2.6% for females and 1.2% for males [25]. While this figure may appear relatively low (even with inclusion of adolescents 10–14 years) compared to adult prevalence rates, it represents a critical public health concern given the developmental vulnerabilities of adolescents, the challenges they face in accessing and adhering to treatment, and the potential for long‐term impacts on their health and well‐being. This statistic underscores the urgent need for adolescent‐responsive HIV services that are tailored to the unique needs of this age group.

This paper details a qualitative study that aimed to explore the perspectives of ALHIV and their caregivers on HIV service delivery and support programs and how these experiences influence adherence to ART in Lusaka district, Zambia. Despite increasing attention to adolescent‐friendly health services, there remains limited studies focused on adolescents and caregiver perspectives of their experience with adolescent‐friendly services (AFS)/differentiated service delivery (DSD) models in HIV service settings and how adolescents and their caregivers perceive and navigate existing HIV services in practice. This study offers several novel contributions: it draws on concurrent dyadic interviews with adolescents and their caregivers within the 2025 service delivery context; it documents disruptions to peer‐support groups linked to funding volatility and it provides insights into challenges at the school–health system interface, including experiences within boarding school environments. By examining the lived experiences of ALHIV aged 10–19 years and their caregivers, this study aimed to inform more responsive, supportive, and context‐specific approaches to improving ART adherence and long‐term treatment outcomes.

## 2. Materials and Methods

### 2.1. Study Setting

This study was conducted in Lusaka, the capital city of Zambia. Lusaka Province encompasses both urban and periurban areas. Adolescent‐specific services are available at tertiary, first‐level, and primary healthcare (PHC) facilities. Tertiary facilities cater to more complex cases, such as treatment failure, coinfections, and mental health concerns. At the first‐level and PHC facilities, adolescents can access services such as HIV and STI care, sexual and reproductive health, counseling for alcohol and substance use, and screening for noncommunicable diseases. These services are made possible through collaborations involving the Ministry of Health, international partners such as PEPFAR through local implementing organizations, and the Global Fund.

### 2.2. Study Design

This exploratory qualitative study used a hybrid inductive‐deductive approach [26] guided by the socioecological model to explore the lived experiences of ALHIV and their caregivers. The design enabled examination of multilevel psychological, social, behavioral, and structural influences on ART adherence, while capturing context‐specific, nuanced insights. Of the participants recruited, a total of 15 adolescent–caregiver dyads were obtained. The remaining adolescents participated without a corresponding caregiver interview because they were above the assent age and attended the health facility independently; therefore, no caregiver was available to form a dyad.

### 2.3. Recruitment and Sampling

Participant recruitment was conducted in collaboration with healthcare providers at the participating facilities. A designated staff member from the ART clinic, familiar with adolescent clients and their caregivers, served as the primary contact for identifying eligible ALHIV aged 10–19 years who were aware of their HIV status and actively receiving ART, along with their primary caregivers. To reduce potential selection bias by clinic staff, eligibility criteria were clearly defined in writing and staff were instructed to approach all adolescents meeting these criteria randomly from the clinic list rather than selecting based on perceived compliance, rapport, or other subjective factors. The research team also cross‐checked participant lists to ensure a representative sample of adolescents across age groups and genders. Eligibility criteria included being on ART, willingness to participate, and the ability to provide informed assent or consent, with caregiver consent required for minors. According to the national guidance, the age of consent for research participation in Zambia is 18 years; therefore, adolescents below 18 years provided written assent in addition to caregiver consent. No Institutional Review Board (IRB) waivers were applied, including for emancipated minors or situations where parental or caregiver permission was not feasible. Using purposive sampling, the study aimed to capture a range of perspectives based on age, gender, and the type of facility (PHC or first‐level). Recruitment took place during routine clinic visits, during which potential participants were given both verbal and written information outlining the study’s purpose, voluntary nature, and confidentiality measures. Those who expressed interest were invited to participate in one‐on‐one interviews, which were scheduled at mutually convenient times in private, adolescent‐friendly rooms within the facilities to ensure comfort and minimal disruption to both clinic operations and participant routines. All participants were assured that their decision to participate or decline would not affect the care they received. No eligible participants declined to participate in the study, and all those approached agreed to be interviewed.

### 2.4. Data Collection and Management

The interview guides were developed based on the research questions related to describing service delivery and support programs for ALHIV on ART. For example, adolescents were asked, “how do you cope with the emotional aspects of living with HIV and adhering to your treatment?”. This was included to elicit insights into psychosocial challenges and coping mechanisms related to ART adherence.

Interview guides were initially developed in English, translated into Nyanja and then back translated into English to verify accuracy. Revisions were made accordingly. The guides were piloted at one health facility to assess clarity before the start of data collection.

Between 20th February and 15th July 2025, a total of 35 individual interviews (IDIs) were conducted with ALHIV (*n* = 20) and their caregivers (*n* = 15). Of these, 15 adolescent–caregiver pairs were interviewed as dyads. The remaining adolescents were above the age requiring caregiver assent and did not attend with a caregiver, hence were interviewed individually.

Thematic saturation was reached at different points for the two participant groups: among caregivers, saturation was observed after the 12th interview, while for adolescents, saturation was reached after the 7th interview for those aged 10–14 years and after the 9th interview for those aged 15–19 years. The ALHIV and their caregivers were drawn from four PHC and two first‐level facilities. All participants were approached in person to explain the purpose of the study and obtain informed consent. A transport reimbursement was provided to all participants to facilitate their participation in the interviews outside their routine schedules. All interviews were conducted in secure, private rooms within the health facilities, free from interruptions, and designed to provide a confidential and adolescent‐friendly environment. Dyad interviews were conducted with attention to confidentiality: some adolescents aged 10–14 years, who were not comfortable being interviewed alone, agreed to be interviewed with their caregivers, while those aged 15–19 years preferred to be interviewed individually and this was respected. During interviews, participants were closely observed for signs of emotional distress. If an adolescent became distressed and needed any psychosocial assistance, referral to facility‐based counselors through established referral pathways were available, ensuring timely and appropriate follow‐up care.

IDIs were carried out in either English or Nyanja, based on participant preference. All interviews were conducted by the first author, who also took notes during each session. These field notes were used for reflection, capturing observations on participants’ nonverbal cues, emotional responses, and contextual details that could complement and enrich the interview transcripts.

All interviews, except one due to the participant’s preference, were audio‐recorded and fully transcribed. For those conducted in Nyanja, the first author translated the transcripts into English to maintain consistency and accuracy. To ensure quality, the first author carefully reviewed each transcript while listening to the corresponding audio recordings to verify accuracy and completeness. Any unclear or ambiguous statements were clarified through careful reflection, and where necessary, notes from the field observations were used to resolve discrepancies. Interviews lasted between 20–30 min with caregivers’ interviews averaging 30 min, older adolescents (15–19 years) averaging 25 min, and younger adolescents (10–14 years) averaging 20 min. No repeat interviews were conducted.

To ensure data security and confidentiality, all transcripts were stored in a password‐protected computer accessible only to the researcher conducting the data collection. Signed consent and assent forms and audio recordings were stored in a locked, secure cabinet to which only the researcher had access. These measures ensured that all sensitive information was safeguarded in accordance with ethical research standards.

### 2.5. Data Analysis

The interview transcripts were uploaded into ATLAS.ti v24 software. Initial (first‐order) coding was conducted inductively [27, 28] to capture emerging patterns and perspectives from participants’ narratives. In the second stage, a deductive approach [29] was applied, using the socioecological model [30] to organize and interpret the data. Specific levels of the model were used as coding categories, resulting in the following themes: intrapersonal (individual), interpersonal, organizational/institutional, and community and structural/policy levels. This allowed for a structured yet flexible analysis of adolescent and caregiver experiences.

The first author initially reviewed four adolescent and two caregiver transcripts (representing 11% of all transcripts) to develop a preliminary codebook. This initial coding framework was then reviewed collaboratively with the second and third authors. As additional transcripts were analyzed, the codebook was iteratively refined to capture emerging themes. This dynamic process involved revising, consolidating, or adding new codes through consensus, ensuring that the analysis remained grounded in the data. Rigor was ensured through collaborative analytic discussions, iterative refinement of the coding framework, and team‐based review of coding decisions. Coding disagreements were handled through collaborative discussion among the research team, during which each coder provided justifications for their interpretations until consensus was reached. This approach aligns with established qualitative practices that emphasize negotiated agreement, reflexive dialog, and collective sense‐making rather than mechanical intercoder reliability [31]. To enhance accuracy and consistency, the research team reread and cross‐checked transcripts against the evolving codes. Pattern coding was subsequently applied to organize initial codes into subthemes, which were then categorized under broader, predefined themes informed by both inductive content analysis and the socioecological model. Data were managed using an Excel matrix structured into three columns: themes, subthemes, and codes. Saturation was considered achieved when further interviews yielded no new insights, themes, or codes. The final analytical matrix synthesized key intrapersonal (individual), interpersonal, organizational/institutional, and community and structural/policy level factors influencing ART adherence among ALHIV, as described by both adolescents and their caregivers. It also highlighted barriers to adherence, available support systems, and participant‐generated recommendations for improving adolescent HIV service delivery (Table 1).

**TABLE 1:** Demographic characteristics of caregivers to the ALHIV.

Characteristics	Females	Males	Total
Caregivers (total)			**15**
Fathers		2	2
Mothers	4		4
Aunts	8		8
Uncles		2	1

*Note:* The bold value provides a distinction of the total from the dissaggregates of care‐givers.

### 2.6. Trustworthiness

The trustworthiness of this study was upheld through rigorous adherence to established qualitative research criteria, namely, credibility, dependability, confirmability, and transferability [32].

Credibility was reinforced through multiple strategies. Interview guides were piloted with ALHIV on ART and their caregivers to ensure cultural appropriateness, clarity, and relevance of the questions. During data collection, iterative probing and clarification were employed to elicit deeper, more nuanced responses. Participants were assured that there were no “right” or “wrong” answers and that their personal experiences were highly valued, creating a safe, open environment that facilitated the sharing of rich, authentic narratives. Member checking was not undertaken, as it was not feasible within the clinical and logistical constraints of the study setting. However, credibility was strengthened through prolonged engagement during interviews and summarizing and asking clarification questions during the interviews and team‐based review of emergent interpretations, which are recognized alternatives in qualitative research. To ensure dependability, a transparent and systematic approach to data collection and analysis was followed. The coding process was thoroughly documented, with the first author conducting the initial coding and coauthors reviewing the coding structure, themes, and analytic decisions to enhance consistency and reliability across the dataset. Confirmability was supported through reflexive practices, including the maintenance of a reflective research journal by the first author. An audit trail was kept documenting methodological decisions, revisions to the analysis, and the rationale behind analytic choices. Analytic debrief meetings were held periodically during which the team reviewed emerging codes, resolved discrepancies, and recorded notable decisions such as redefining code boundaries, merging overlapping categories, and adjusting theme structures. Transferability was facilitated by providing rich, detailed descriptions of the study setting, participant characteristics, and research procedures. These contextual insights enable readers to assess the relevance and applicability of the findings to similar contexts. Peer debriefing with experienced qualitative researchers was conducted throughout the study to critically assess emerging findings, challenge assumptions, and enhance analytical rigor by incorporating alternative interpretations.

Finally, the study adhered to key components of the Consolidated Criteria for Reporting Qualitative Research (COREQ) checklist [33], ensuring comprehensive and transparent reporting of methodological processes and findings.

### 2.7. Ethical Approval

Ethics clearance was obtained from the University of the Western Cape Biomedical Research Ethics Committee (reference number: BM24/3/4) , the Mulungushi University School of Medicine Ethics Committee (reference number: SMHS‐MU2‐2024‐04), and the Zambia National Health Research Authority (reference number: NHRA1186/15/05/2024). Approval for the study and access to health facilities were obtained from the Zambia Ministry of Health (reference number: MH/101/22/3). The researchers sought verbal and written consent from all study participants before participation in the study. Pseudonyms were used to identify study participants in the transcription of IDIs. In addition, participants consented to the publishing of their responses if their identities were kept anonymous. No personal information was collected from participants during the research process.

## 3. Findings

Table 2 presents the demographic characteristics of the ALHIV who participated in the study, along with the caregiver participants of ALHIV. A total of 35 participants were interviewed, comprising 20 adolescents and 15 caregivers. Among the adolescents, 16 were currently enrolled in school (primary or secondary) and 1 was in tertiary institutions (college/university), while 3 had recently completed secondary education and were awaiting entry into tertiary institutions. All younger adolescents (aged 10–14 years) were still in school. The caregivers included mothers (*n* = 4), fathers (*n* = 2), aunts (*n* = 8), and agrandmother (*n* = 1).

**TABLE 2:** Demographic characteristics of ALHIV.

Characteristics	Females	Males	Total
Adolescents (total)	8	12	**20**
10–14	3	6	9
15–19	5	6	11
In school	7	10	17

*Note:* The bold value provides a distinction of the total from the dissaggregates of age and those in school.

Five main themes emerged from the analysis of the interview data: intrapersonal (individual), interpersonal, organizational/institutional, community, and structural/policy‐level factors influencing ART adherence and cross‐cutting recommendations (Table 3). Each theme is described alongside its corresponding subthemes, with illustrative verbatim quotes included where appropriate to support the authenticity and credibility of the coding process.

**TABLE 3:** Intrapersonal (individual), interpersonal, organizational/institutional, community, and structural/policy‐level factors influencing ART adherence and cross‐cutting recommendations for improved service delivery targeting ALHIV and their caregivers.

Theme	Subtheme	Codes: adolescents and caregivers
Intrapersonal (individual) level	Psychological factors	
	Emotional burden (impact of diagnosis and chronic illness)	• Shock • Grief • Regret • Chronic condition fatigue • Emotional distress • Fear of the future • Denial • Emotional exhaustion • Isolation within the family • Fear of treatment discontinuation
	Internalized stigma and fear	• Self‐stigma • Peer influence and stigma • Fear of death • Secrecy around HIV status • Fear of disclosure
	Adaptation and acceptance (response)	• Acceptance of HIV status • Understanding HIV status • Resilience
	Positive coping strategies (response)	• Spirituality • Reliance on prayer • Distraction through work • Ongoing counseling • Strong internal motivation
	Behavioral factors	
	Adherence barriers	• Initial fear of HIV transmission to others • School‐related scheduling challenges • Lack of structured routines • Disruption in early caregiving practices
	Routine and medication management	• Adherence routines • Time adjustments • Structured routine • Medication timing adjusted for school • Bedtime routines
	Technology and reminder use	• Alarm systems • Phone alarms • Discreet reminders
	Health literacy and ART understanding (support/response)	• Understanding the importance of timing • Awareness of side effects • Understanding ART necessity and longevity • Education at home; knowledge of viral load monitoring • Knowledge reinforcement from school

Interpersonal level	Social factors	
	External stigma and disclosure challenges	• Disclosure challenges • Emotional support post‐disclosure • Limited household disclosure • Secrecy due to social stigma
	Family‐based support (response)	• Parental support • Caregiver support • Sibling/relative encouragement • Family involvement in ART
	Peer and community support (response)	• Peer support groups • Encouragement by neighbours • Clinic and church support
	Healthcare relationships and communication (support/response)	• Positive clinic experiences • Supportive healthcare workers • Support for disclosure • Adolescent‐focused services

Organizational/institutional level	Health system access and service delivery	
	Clinic access and service barriers	• Clinic inefficiencies • Delayed services • Drug access challenges
	Clinic access and service use (response)	• Clinic efficiency • DSD program access • Adolescent‐friendly services • Reliable drug access • Clinic‐to‐clinic coordination

Community level	Social factors	
	Social stigma and disclosure challenges	• Disclosure challenges • Emotional distress post‐disclosure • Limited household disclosure • Stigma avoidance
	Community support structures (response)	• Emotional support post‐disclosure • Community encouragement

Structural/policy level	Socioeconomic and structural determinants of care	
	Socioeconomic and structural barriers	• Financial strain • School material support needs • Transport issues • Borrowing money for clinic access • Living conditions limiting privacy
	Structural support systems (response)	• Transport facilitation • Financial assistance • Social protection • School support

Cross‐cutting recommendations	System‐level responses	• Peer support networks • Youth‐friendly community spaces • Relationship and life guidance • Support for health workers to implement adolescent‐friendly services • Mental health services • Improved communication strategies • Proactive follow‐up systems • Health education sessions • Clinic‐based nutritional support • Reactivate inactive support groups

### 3.1. Intrapersonal (Individual) Level

#### 3.1.1. Psychosocial Factors

Adolescents and caregivers described a range of psychological experiences related to HIV diagnosis, lifelong treatment, and the emotional toll of living with a chronic condition. These experiences were grouped under four key subthemes: emotional burden, internalized stigma, and fear (barriers), followed by adaptation and positive coping strategies (responses).

##### 3.1.1.1. Emotional Burden (Impact of Diagnosis and Chronic Illness) (Barrier)

Both adolescents and caregivers recounted strong emotional reactions to HIV status disclosure, including shock, grief, and regret. Several participants described an overwhelming sense of emotional exhaustion and distress, often triggered by the realization of the lifelong nature of the condition.

*“The moment I reached 16, that’s when they confronted me… and it was hard for me to just accept that… I even started throwing the medications.” (Adolescent, Male, 16 years)*

*“It has (child’s diagnosis) affected me a lot. I always think, from the other side, I think a lot, I go beyond. I think life is not fair.” (Caregiver, 01, aunt)*

A few adolescents, however, reported minimal emotional distress, stating they had simply accepted their status as a part of life. One adolescent noted, “*I just live a normal life because I mostly pretend that I’m not sick.”* (adolescent, female, 13 years).

##### 3.1.1.2. Internalized Stigma and Fear (Barrier)

A prominent theme across narratives was the presence of self‐stigma, peer‐induced stigma, and internalized fear. Adolescents described keeping their HIV status a secret, even from close friends, due to the fear of judgment, rejection, or being labeled.

*“Because it’s for my own safety and privacy.” (Adolescent, Male, 15 years)*

This was reaffirmed by caregivers, who often reported concealing the adolescent’s status from extended family and community members to protect them from stigma and discrimination.

*“Only me, father and brother know. Community doesn’t.” (Caregiver, 10, Aunt).*

Some adolescents also described the negative impact of peer influence and stigma, particularly in boarding school or community settings where disclosure could lead to gossip or isolation.

*“Mostly being at school… I do it (take medication) secretly. I’ve thought about dating with four guys. The other ones, they just left me… I told them (HIV status), they accepted, yes. And then they just decided to go.” (Adolescent, Female, 19 years).*

##### 3.1.1.3. Adaptation and Acceptance (Response to Emotional Burden and Stigma)

Despite initial emotional struggles, many adolescents demonstrated varying degrees of adaptation over time. This process often began with the acceptance and understanding of their HIV status, particularly following disclosure. Some adolescents developed a sense of resilience, drawing strength from consistent ART adherence and support systems.

*“I just let it go. I remind myself that there are many people in the world who are living with HIV and still living normal lives.” (Adolescent, Male, 15 years).*

Conversely, a few adolescents described a prolonged struggle with adaptation, where acceptance was not fully achieved. For example, one participant’s goal was not to manage the condition but to stop treatment entirely, stating, *“I want to come out of the medication. I just want to stop and stop coming here. I’m tired.”* (adolescent, female, 13 years).

##### 3.1.1.4. Positive Coping Strategies (Response)

In navigating these emotional challenges, adolescents adopted various coping mechanisms. Spirituality and prayer were frequently mentioned as sources of comfort and strength, alongside distraction through work, strong internal motivation, and ongoing counseling.

*“I encourage myself, you know, by putting God first… through God’s word.” (Adolescent, Female, 19 years).*

Caregivers also highlighted faith‐based coping, noting that prayer and trust in god helped both them and the adolescents manage the emotional burden of living with HIV.

*“I simply trust that God will help us. I surrender everything to Him in prayer, asking for guidance and direction.” (Caregiver, 02, Aunt).*

Ongoing counseling, provided at clinics or through peer support, played a critical role in fostering internal motivation and helping adolescents reframe their understanding of living with HIV.

*“I usually look out for people in the same situation as mine, so that I get encouraged to adhere to my medicine.” (Adolescent, Female, 19 years).*

#### 3.1.2. Behavioral Factors

In addition to psychological and social influences, adolescents’ day‐to‐day behaviors and routines played a crucial role in shaping their adherence to ART. These experiences were categorized into four subthemes: adherence barriers, routine and medication management, technology and reminder use, and health literacy and ART understanding.

##### 3.1.2.1. Adherence Barriers

Despite these efforts, several barriers disrupted adherence routines. Some adolescents recalled an initial fear of transmitting HIV to others, particularly before fully understanding the virus.

*“People always say that if you meet with someone who has HIV, you might get infected. Or just having them around with you, you might even get infected.” (Adolescent, Male, 15 years).*

Others faced school‐related conflicts, such as being in boarding school, early departure times, or after‐school activities that interfered with medication schedules.

*“I was at school (boarding) and it was far… Yes, I missed taking my medication sometimes.” (Adolescent, Female, 18 years).*

Inconsistent caregiving practices, especially among those who changed caregivers frequently or had limited supervision, further complicated routine formation.

*“My sister’s mom, she doesn’t know and I made an excuse to come back (home).” (Adolescent, Female, 17 years).*

##### 3.1.2.2. Routine and Medication Management (Response)

In response to these challenges, adolescents and caregivers described developing structured routines to support consistent medication use. This included adjusting medication times around school schedules, setting bedtime routines, and creating daily adherence habits.

*“I always take my medicine around 6 hours. I go to school in the morning.” (Adolescent, Female, 14 years).*

*“Since all of us are on ART, we use an alarm for 8 PM and take medication at the same time.” (Caregiver, 03, Father).*

A few adolescents, however, reported significant and ongoing difficulties in establishing a stable routine. One participant cited their living situation as a major barrier, stating

*“we are too many at our place and it is difficult for me to take my medicine.” (Adolescent, Male, 14 years).*

##### 3.1.2.3. Technology and Reminder Use (Response)

To further strengthen these routines, both adolescents and caregivers employed a range of reminder tools, most commonly phone alarms and discreet alert systems. Adolescents appreciated the privacy and autonomy that such tools provided, especially in environments where stigma or disclosure concerns were present.

*“I adjust the timer occasionally, so my friends don’t notice a pattern.” (Adolescent, Female, 16 years).*

These technologies helped reduce forgetfulness and empowered adolescents to take ownership of their medication schedule, particularly in contexts where adult supervision was limited or inconsistent.

*“Alarm is one of the strategies I had put to make sure that I adhere to my medicine.” (Adolescent, Female, 19 years).*

##### 3.1.2.4. Health Literacy and ART Understanding (Support/Response)

Many adolescents demonstrated a strong understanding of the importance of ART timing, the long‐term necessity of treatment, and the awareness of side effects, including knowledge of viral load monitoring and reinforcement of information at home and school.

*“By then I was a child. I never understood why I was taking medicine. But when my parents sat me down and told me… now I know why.” (Adolescent, Female, 14 years).*

Adolescents who were aware of viral load monitoring and the broader purpose of ART expressed more confidence in maintaining adherence.

*“I want to prevent the viruses from spreading around the body, which may help me in the future and I will have to stay a longer life.” (Adolescent, Male, 15 years).*

Both adolescents and caregivers emphasized that post‐disclosure education, including repeated, age‐appropriate explanations, was crucial for enabling informed adherence and reducing fear or confusion.

*“She has now accepted and is adherent.” (Caregiver 04, Mother).*

### 3.2. Interpersonal Level

#### 3.2.1. Social Factors

Adolescents’ and caregivers’ accounts also highlighted the influence of social relationships and support systems on treatment adherence and emotional well‐being. These were categorized under four subthemes: external stigma and disclosure challenges, family‐based support, peer and community support, and healthcare relationships and communication.

##### 3.2.1.1. External Stigma and Disclosure Challenges

Despite strong interpersonal support in many cases, external stigma and disclosure remained a persistent challenge. Adolescents and caregivers described the need to conceal their status from peers, extended family, and school environments due to the fear of social rejection.

*“I’ve always told them (caregivers) that I should not tell people. Many people might not accept you the way you are.” (Adolescent, Male, 15 years).*

*“No one knows about her HIV status apart from her grandmother, who is my mother and my two sisters. But the outside and the extended family, we have not yet disclosed anything.” (Caregiver, 01, Uncle).*

Some adolescents avoided taking their medication in front of others or skipped doses altogether to avoid suspicion. Both adolescents and caregivers described careful secrecy as a strategy to protect the adolescent from discrimination.

*“Mostly being at school, friends in the room. I’m the only one taking medication. I do it secretly. My friends don’t know.” (Adolescent, Female, 19).*

At the same time, a few adolescents reported that stigma did not directly affect their adherence, largely because they managed it through strict secrecy.

*“As for me, I think I was not stigmatized because I kept it to myself.” (Adolescent, Male, 16 years)*

##### 3.2.1.2. Family‐Based Support (Response)

Support from family, particularly parents, caregivers, and siblings emerged as a cornerstone of adherence and emotional stability. Adolescents described consistent reminders from caregivers to take their medication and attend clinic appointments.

*“With encouragement from my parents, I make it a point to wake up early in the morning to go to the clinic to pick up my medication.” (Adolescent, Female, 13 years).*

This support was reaffirmed by caregivers, who reported actively monitoring adherence, offering reminders, and coordinating clinic visits to ensure continuity of care.

*“We usually remind him. Sometimes we’ll call him from another room… then remind him to take his medicine.” (Caregiver, 08, Aunt).*

However, a few adolescents described disrupted or inconsistent family support, particularly when staying away from their primary caregivers, which negatively affected adherence routines.

*“There was this period whereby I went to visit my auntie’s place… I was used to dad telling me, okay, it’s time for medication… since I was tired, it slipped my mind that I’m supposed to take my medication.” (Adolescent, Male, 16 years)*

##### 3.2.1.3. Peer and Community Support

Beyond the household, peer and community‐level support played an equally vital role. Adolescents who participated in peer support groups reported feeling more understood and less alone in their experiences. This was re‐affirmed by caregivers.

*“After disclosure and attending the support group, he understood he wasn’t alone. That support group has helped him a lot.” (Caregiver, 08, Aunt).*

Some caregivers mentioned how neighbors or church members, aware of the adolescent’s status, offered informal counseling and encouragement. These community contacts helped reinforce key adherence behaviors and reduced the shame associated with HIV.

*“I have confided in one elderly neighbor who assists and so at times when she has challenges, she assists in counselling.” (Caregiver, 03, Father).*

In contrast, some adolescents reported avoiding peer or community support due to fear of disclosure and stigma, resulting in limited social support beyond the household.

*“I actually don’t think about disclosing because I feel as though they won’t be close to me anymore because they don’t understand what HIV is.” (Adolescent, Male, 16 years)*

##### 3.2.1.4. Healthcare Relationships and Communication

Interactions with the healthcare system significantly influenced adolescents’ treatment journeys. Adolescents often referenced positive relationships with healthcare workers, who were described as nonjudgmental, patient, and understanding.

*“The health workers tell me how important it is to take my medicine on time and never stop. They explain what can happen if I don’t take it.” (Adolescent, Female, 16 years).*

This was reaffirmed by caregivers, who also highlighted the supportive nature of healthcare workers, noting their approachability, effective communication, and role in promoting adherence and emotional well‐being.

*“The health workers are good. They always call us, even for appointments. They call three days before and even on the appointment day, they follow up to check why we missed it.” (Caregiver, 07, Aunt).*

Healthcare providers also played a key role in supporting disclosure, both by guiding caregivers through the process and by reinforcing health literacy among adolescents’ post‐disclosure. These respectful and age‐appropriate interactions enhanced trust and encouraged adolescents to attend clinic visits more consistently.

*“The health workers advised us. One of the staff at this clinic encouraged me to start explaining to him why he takes those drugs.” (Caregiver, 08, Aunt).*

### 3.3. Organizational/Institutional Level

#### 3.3.1. Health System Access and Service Delivery

Health system access and service delivery shaped adolescents’ ability to engage consistently with HIV care. Participants described how clinic organization, service efficiency, and drug availability influenced appointment attendance and treatment adherence. While some experienced structural and scheduling barriers, others benefited from coordinated AFSs that supported continuity of care. These accounts highlight the health system as both a barrier and an enabler of sustained engagement in treatment.

##### 3.3.1.1. Clinic Access and Service Barriers

Some adolescents reported organizational barriers, particularly clinic operating hours conflicting with school schedules.

*“Since the school knocks off late, sometimes you find that you are punished or kept for group studies and you are late to reach the clinic.” (Adolescent, Male, 19 years)*

##### 3.3.1.2. Clinic Access and Service Use (Response)

Access to adolescent‐friendly health services emerged as a key support system promoting adherence. Adolescents described the importance of well‐organized and coordinated clinic visits where health workers were supportive, respectful, and responsive to their unique needs. DSD models such as multimonth ART refills were particularly valued for reducing the burden of frequent clinic visits and supporting sustained engagement in care.

*“It did not have any impact (delayed drug refill) because they gave me (drug supply) for six months and I still had some tablets.” (Adolescent, Male, 14 years).*

*“When I inform them that I will be travelling, they communicate with the clinic in the area I am visiting.” (Adolescent, Female, 16 years).*

### 3.4. Community Level

#### 3.4.1. Social Stigma and Disclosure Challenges

Social stigma and fears around disclosure emerged as important factors shaping adolescents’ daily experiences of living with HIV. Participants described carefully managing their treatment routines to avoid raising suspicion among peers, reflecting concerns about being judged or treated differently if their status became known. One adolescent explained,

*“I adjust the timer occasionally, so my friends don’t notice a pattern. If the alarm always rings at the same time, they might become suspicious.” (Adolescent, Female, 19 years).*

Disclosure was largely limited to trusted family members, with some adolescents intentionally keeping their status private beyond the household. As one participant noted,

“I haven’t disclosed it outside of my family.”

#### 3.4.2. Community Support Structures (Response)

Some adolescents described receiving encouragement and emotional support from community members post‐disclosure.

“I used to come for the Youth Friendly Space… it does help.”

### 3.5. Structural/Policy Level

#### 3.5.1. Socioeconomic and Structural Determinants of Care

##### 3.5.1.1. Socioeconomic and Structural Barriers

Across the interviews, adolescents and caregivers highlighted the pervasive role of socioeconomic and structural constraints in shaping ART adherence and access to care. Financial strain, school material needs, transport challenges, and living conditions limiting privacy affected care access.

Financial strain was a common theme, particularly among caregivers who struggled to meet daily household needs while also supporting the adolescent’s medical care.

*“It was difficult, I spent a lot of money… It was really difficult being the only person working in the family.” (Caregiver, 01, Uncle).*

Some adolescents reported lacking essential materials, such as notebooks or uniforms, which not only affected their education but also contributed to low self‐esteem and reduced motivation.

*“Things for school like tuition fees, books and other school-related needs.” (Adolescent, Male, 14 years).*

Caregivers to adolescents living in periurban areas often had to borrow money or walk long distances to reach ART clinics. These logistical constraints sometimes led to missed appointments or inconsistent medication refills.

*“At times, I would go a day or two later to the clinic for his appointment. I have to get credit from friends.” (Caregiver, 10, Aunt).*

In addition to household financial challenges, structural barriers such as permission from the school to attend appointments, transportation difficulties, and living conditions limiting privacy significantly affected clinic access.

*“Sometimes, as you know, these private schools don’t give permission easily and only a few of them understand.” (Caregiver, 01, Uncle).*

##### 3.5.1.2. Structural Support Systems (Response)

Some adolescents described successfully navigating these barriers through planning and caregiver support.

*“I try to visit the clinic on weekends before my medication runs out.” (Adolescent, Female, 16 years)*

### 3.6. Cross‐Cutting Recommendations

During the interviews, both adolescents and caregivers provided valuable insights into potential improvements that could enhance ART adherence and overall well‐being for ALHIV. These recommendations fell into two broad subthemes: addressing social support needs and enhancements in service delivery.

#### 3.6.1. System Level Responses

Participants expressed a strong desire for greater access to peer support networks where adolescents could interact with others facing similar challenges. These spaces were seen not only as forums for encouragement but also as platforms for learning practical coping strategies. Several adolescents noted that youth‐friendly community spaces would help reduce isolation and offer safe environments for social interaction without stigma.

*“I wanted to make up a group of helping my friends who are having difficulties in taking drugs, like here.” (Adolescent, Male, 19 years).*

In addition to peer connections, participants emphasized the need for guidance on relationships. Adolescents expressed that support should extend beyond ART to include broader psychosocial and developmental support, particularly around managing romantic relationships and aspirations for the future.

*“We don’t know what to do in the future (in terms of relationships).” (Adolescent, Female, 17 years).*

Caregivers and adolescents alike highlighted opportunities for improving the health system’s responsiveness to adolescent needs. A key recommendation was to strengthen AFSs by providing targeted support and capacity‐building for health workers. This included adolescent communication, confidentiality, and empathy, all of which were seen as vital to maintaining engagement in care.

*“Maybe by calling and reaching out (communication) to our parents.” (Adolescent, Female, 13 years).*

Adolescents indirectly recommended the integration of mental health services into routine HIV care, recognizing the emotional burden and stress adolescents carry.

*“There are those patients that undergo stress and are unable to drink medicines because of their stress and due to emotional problems… they are supposed to take them, like, lightly.” (Adolescent, Female, 16 years).*

Improved communication strategies, such as active involvement of adolescents in treatment discussions, were suggested to promote health literacy and empowerment.

*“I think maybe when you enter there with your parents, it’s best if it’s just a patient (without parent) with a doctor to talk. At least I can be freer.” (Adolescent, Female, 13 years).*

Some adolescents called for proactive follow‐up systems, including home visits or phone check‐ins for adolescents missing appointments.

*“They should be calling adolescents to check on how they’re doing.” (Adolescent, Female, 19 years).*

Others emphasized the importance of clinic‐based health education sessions, not only for adolescents but also for caregivers, to improve shared understanding of ART and treatment goals.

*“Creating support groups… talking to the parents… counselling sessions with the parents explaining why it’s important.” (Adolescent, Male, 16 years).*

Additional suggestions included nutritional support at clinics.

*“They should support us more. For example, they could provide snacks or food. We come early, around 8 AM and sometimes we haven’t eaten anything.” (Adolescent, Male, 14 years).*

Furthermore, the reactivation of inactive adolescent support groups was suggested, as many adolescents felt these had previously served as valuable sources of psychosocial support. These groups had been closed following a stop work order issued during the freeze of PEPFAR funding, which led to staff unavailability and disrupted service continuity.

*“I would like the meetings to start again. They used to teach us a lot of things. Now they’re no longer there.” (Adolescent, Female, 17 years).*

## 4. Discussion

This study highlights the complex and interconnected intrapersonal (individual), interpersonal, organizational/institutional, community, and structural/policy level factors shaping ART adherence among ALHIV in Lusaka. Through the voices of adolescents and their caregivers, our findings reveal that while many ALHIV experience emotional distress, stigma, and structural hardship, they also demonstrate resilience, adaptability, and a strong desire for support in navigating lifelong treatment. Importantly, the findings also illustrate variability in adolescents’ experiences, with some reporting minimal emotional distress or successfully managing stigma through secrecy, while others struggled with acceptance and adherence, underscoring the need for differentiated, adolescent‐centered responses.

At the intrapersonal (individual) level, adolescents’ experiences were shaped by interconnected psychological and behavioral factors that influenced their engagement with HIV treatment and adherence. Psychological experiences surrounding diagnosis and treatment were central to adolescent narratives. Emotional reactions such as grief, anxiety, and internalized stigma echoed findings from other Sub‐Saharan African contexts, where adolescents often struggle with disclosure, self‐acceptance, and treatment fatigue [34]. However, our study also found that with time, adolescents adapted through positive coping strategies, including spirituality, acceptance, and supportive counseling [35]. At the same time, some adolescents reported prolonged difficulty accepting lifelong treatment or expressed a desire to discontinue medication, highlighting the ongoing need for mental health and adherence support beyond initial disclosure. This psychological adjustment translated into behavioral responses, as behavioral factors such as routine formation, health literacy, and the use of reminder tools such as phone alarms were pivotal in supporting adherence. Adolescents who understood the importance of ART and could incorporate treatment into daily life demonstrated greater consistency. These findings are also reported in similar settings where reminder tools such as alarms, structured routine, and health literacy were critical facilitators of adherence [36]. Our findings further suggest that early caregiving practices and stability in supervision influence routine formation, indicating that adherence behaviors are shaped not only by individual motivation but also by caregiving environments over time. In addition, individual adherence behaviors were supported by service delivery models that reduced treatment burden, notably, DSD approaches, such as multimonth dispensing and clinic‐to‐clinic referrals, facilitated adherence by accommodating adolescents’ schooling and travel patterns. These approaches are recommended in local policy and were implemented in the health facilities [37, 38]. These flexible models should be scaled up, particularly for mobile or school‐going adolescents.

Consistent with previous research, interpersonal support, particularly from caregivers, emerged as a critical facilitator of adherence [11, 39]. From a social ecological model (SEM) perspective, these findings highlight the central role of interpersonal relationships in shaping adolescents’ treatment behaviors. Adolescents who described close, communicative relationships with family members were more likely to establish consistent medication routines, suggesting that practical support such as reminders, encouragement, and active family involvement in ART can buffer against adherence challenges during adolescence [15, 40]. However, disruptions in caregiving arrangements or limited disclosure within households sometimes weakened these support systems and contributed to missed doses or inconsistent routines. Peer and community support, including participation in peer support groups, encouragement from neighbors, and support within clinic and church settings, as well as engagement with empathetic healthcare workers, further contributed to adolescents’ emotional well‐being and motivation to remain on treatment [41]. Conversely, some adolescents avoided peer engagement due to fear of stigma and disclosure, resulting in social isolation and limited support beyond immediate caregivers. Importantly, positive healthcare relationships, adolescent‐focused services, and supportive communication around appear to strengthen trust and normalize long‐term engagement in care [42]. These findings reinforce the importance of family‐centered, peer‐led, and relationally supportive interventions at the interpersonal level of the SEM, underscoring their relevance for strengthening adolescent HIV care beyond individual‐level approaches.

Stigma, both internalized and external, remained a persistent barrier. At the community level of the SEM, external stigma and prevailing social norms shape adolescents’ disclosure decisions and access to emotional and social support [43, 44]. Adolescents in the current study concealed their status due to fear of rejection, particularly in school and peer settings, reflecting stigma avoidance strategies within community and social environments. Caregivers also described limiting disclosure within families and communities to protect adolescents from discrimination, resulting in limited household disclosure and constrained emotional support post‐disclosure. This secrecy, while protective, often restricted access to broader support networks embedded within communities. Notably, some adolescents reported that maintaining secrecy enabled adherence by avoiding stigma, indicating that disclosure decisions are highly contextual and require individualized counseling approaches. These insights align with literature suggesting that interventions to reduce HIV‐related stigma must extend beyond clinical settings and address household and community‐level attitudes that sustain stigma and normalize secrecy [44–46].

Importantly, our findings show that the school environment itself functions as a significant institutional‐level bottleneck. Within the SEM, this reflects organizational and institutional constraints that shape adolescents’ ability to access and utilize adherence support systems. Adolescents reported difficulties taking medication at school due to rigid schedules, limited privacy, and fear of inadvertent disclosure [47], which mirror broader challenges related to service accessibility, institutional routines, and the absence of adolescent‐friendly systems within nonhealth settings. School‐related pressures, including boarding arrangements, permission requirements for clinic visits, and competing academic demands, further disrupted adherence routines for some adolescents. These challenges underscore the need for stronger coordination between the education and health sectors to ensure that school policies are aligned with adolescent‐responsive and DSD strategies that complement clinic‐based adherence support such as flexible medication pick‐up times, discreet storage options for ART, and school‐based support systems that protect confidentiality. Such alignment is particularly important given the role of clinics in providing efficient, coordinated services, reliable drug access, and DSD models tailored to adolescents. Strengthening this intersectoral collaboration is critical for realizing the intent of national adolescent health policies, which emphasize supportive school environments as a core enabler of adherence and long‐term retention in care [42, 48, 49]. These findings underscore the necessity of holistic, multisectoral responses at the organizational and institutional level of the SEM.

Structural and socioeconomic constraints, particularly transport challenges, school conflicts, and poverty, undermined adherence for many adolescents in the current study. At the structural and policy level of the SEM, these barriers reflect broader socioeconomic conditions and systemic inequities that shape adolescents’ capacity to remain engaged in care. Financial insecurity limited access to school materials and clinic visits, often requiring adolescents or caregivers to borrow money for transport, while overcrowded homes eroded privacy and constrained safe spaces for medication storage and dosing. Living conditions that limited privacy and frequent caregiver economic stress further influenced adolescents’ emotional well‐being and motivation to adhere to treatment. These findings align with evidence from Kenya and a broader study in southern Africa that similarly highlighted structural barriers to ART adherence among adolescents [47, 50], underscoring the need for policy‐level interventions that integrate social protection, transport support, and adolescent‐sensitive service delivery within national HIV programmes.

Participants offered a range of actionable recommendations that can be organized into three interconnected programmatic priorities: restoring disrupted services, strengthening existing practices, and introducing new or innovative elements to enhance adolescent HIV care. First, both adolescents and caregivers emphasized the need to restore services that had weakened over time, particularly the reactivation of adolescent and caregiver support groups and more consistent counseling, especially following disclosure and reliable implementation of DSD practices such as predictable multimonth dispensing and timely clinic‐to‐clinic referrals [11, 51, 52]. Restoring these core components was viewed as critical to rebuilding trust, continuity, and psychosocial support. Notably, adolescents called for more autonomy and involvement in treatment discussions, reflecting a need to transition toward adolescent‐centered models of care that build self‐management skills [51, 53].

In addition, participants highlighted opportunities to strengthen existing practices that already form part of routine care. These included improving provider–adolescent communication through more supportive, adolescent‐friendly engagement, enhancing caregiver involvement by improving their treatment literacy and communication skills and reinforcing ongoing health education sessions to build adolescents’ understanding of ART and self‐management [42, 48, 49]. Strengthening existing DSD approaches by more consistently aligning appointment schedules with school demands, as well as integrating routine mental health screening into clinic visits, was also seen as essential for sustaining adherence [54, 55]. Participants also emphasized the need for proactive follow‐up systems, such as phone calls or outreach for missed appointments and expanded caregiver‐focused education to support shared responsibility for adherence.

Finally, participants proposed several innovative strategies that could extend current service models and better respond to adolescents’ evolving needs. These included creating structured platforms for adolescent involvement such as youth advisory groups to promote autonomy in treatment decision‐making; establishing more formalized coordination between health facilities and schools to minimize schooling disruptions and support discreet medication‐taking, expanding digital adherence tools such as SMS reminders or interactive messaging platforms, and enhancing adolescent‐friendly clinic environments through flexible service hours and dedicated spaces [49, 56, 57]. Additional suggestions included integrating mental health services into routine HIV care, providing clinic‐based nutritional support for adolescents attending appointments, strengthening youth‐friendly community safe spaces, and ensuring continuity of peer support platforms disrupted by funding or service interruptions.

## 5. Limitations of the Study

This study had some limitations. First, as a qualitative study relying on IDIs, the findings are based on self‐reported experiences and perceptions, which may be subject to recall bias or social desirability bias, particularly when discussing sensitive topics such as adherence, stigma, or household dynamics. Second, younger adolescents (ages 10–14 years) had difficulties articulating their experiences with the same depth as older participants, potentially limiting the richness of data from this subgroup. Although caregivers helped provide additional context, their perspectives may not fully capture the experiences of the adolescents themselves. Third, participation was limited to adolescents and caregivers actively engaged in HIV care, which may exclude the voices of those who have disengaged from treatment and whose perspectives might reveal additional or more severe barriers to adherence. The findings may therefore not be generalizable to all ALHIV, particularly those lost to follow‐up. Additionally, information on adolescents’ schooling status (e.g., boarding versus day school) was not collected, limiting the study’s ability to fully interpret how school environments and routines may influence adherence and access to care. Future research should incorporate the schooling context, given its known relevance to adolescent health behaviors. Finally, interviews were conducted in facility settings, which, despite efforts to ensure privacy, may have influenced participants’ willingness to speak freely. Nevertheless, trust‐building measures and the use of adolescent‐friendly interview techniques helped foster open dialog.

## 6. Conclusion

Adherence among ALHIV in Lusaka is shaped by a dynamic interplay of psychological, social, behavioral, and structural factors. Interventions must move beyond individual‐level strategies to consider broader family, community, and system‐level enablers and barriers. Strengthening psychosocial support, enhancing AFS delivery, and addressing stigma and poverty are key priorities for improving outcomes in this population.

**Nomenclature**
ALHIV: Adolescents living with HIV
ART: Antiretroviral therapy
COREQ: Consolidated Criteria for Reporting Qualitative Research
HIV: Human immunodeficiency virus
IDIs: In‐depth interviews
IRB: Institutional Review Board
PEPFAR: President’s Emergency Plan for AIDS Relief
PHC: Primary health care
ZDHS: Zambia Demographic and Health Survey

## Funding

This work is based on the research supported by the Belgian Directorate‐General for Development Cooperation, through its framework agreement with the Institute of Tropical Medicine (Grant Ref: FA5 DGD‐ITM 2022‐2026).

## Conflicts of Interest

The authors declare no conflicts of interest.

## Data Availability

The data that support the findings of this study are available from the corresponding author upon reasonable request.
